# On-Orbit Robotic Grasping of a Spent Rocket Stage: Grasp Stability Analysis and Experimental Results

**DOI:** 10.3389/frobt.2021.652681

**Published:** 2021-06-17

**Authors:** Nikos Mavrakis, Zhou Hao, Yang Gao

**Affiliations:** STAR-Lab, Surrey Space Centre, University of Surrey, Guildford, United Kingdom

**Keywords:** robotic grasping, space debris removal, on-orbit servicing, grasp stability, orbital robotics

## Abstract

The increased complexity of the tasks that on-orbit robots have to undertake has led to an increased need for manipulation dexterity. Space robots can become more dexterous by adopting grasping and manipulation methodologies and algorithms from terrestrial robots. In this paper, we present a novel methodology for evaluating the stability of a robotic grasp that captures a piece of space debris, a spent rocket stage. We calculate the Intrinsic Stiffness Matrix of a 2-fingered grasp on the surface of an Apogee Kick Motor nozzle and create a stability metric that is a function of the local contact curvature, material properties, applied force, and target mass. We evaluate the efficacy of the stability metric in a simulation and two real robot experiments. The subject of all experiments is a chasing robot that needs to capture a target AKM and pull it back towards the chaser body. In the V-REP simulator, we evaluate four grasping points on three AKM models, over three pulling profiles, using three physics engines. We also use a real robotic testbed with the capability of emulating an approaching robot and a weightless AKM target to evaluate our method over 11 grasps and three pulling profiles. Finally, we perform a sensitivity analysis to demonstrate how a variation on the grasping parameters affects grasp stability. The results of all experiments suggest that the grasp can be stable under slow pulling profiles, with successful pulling for all targets. The presented work offers an alternative way of capturing orbital targets and a novel example of how terrestrial robotic grasping methodologies could be extended to orbital activities.

## 1 Introduction

Robots are deployed more and more in space applications, and their operations require various levels of autonomy. Current research directions, market forecasts, and space agencies’ policies indicate that in the future robotic arms will be increasingly used in the novel applications of on-orbit servicing and space debris removal, and in-space manufacturing. The main operation that robotic arms need to perform in orbit has been docking and berthing with both cooperative and non-cooperative targets. Docking typically requires a mechanical grappling component on the robot’s end-effector, and a matching passive interface on the target. A variety of interfaces have been developed for this purpose [Aikenhead et al. (1983), Goeller et al. (2012), Medina et al. (2017), Wenzel et al. (2017), Li et al. (2019)]. Such an interface may not always exist, as it is the case with older payloads and space debris. This calls for the design of more elaborate capturing methods, with a comprehensive review on methods for capturing orbital targets given by Shan et al. (2016). In this paper, we focus on the usage of robotic fingers rather than actuating mechanisms such as harpoons, nets, etc. We also consider a target not equipped with a passive interface for docking.

In the case where a passive interface is not present, a chasing spacecraft has to capture the target from another structural part. Early robotic missions, such as the ETS-VII used robotic clamps that grasp the target by existing handles, and detected the handle position with visual markers [Inaba and Oda (2000)]. The NASA Astrobee assisting free-flying robot has demonstrated handle detection and grasping on the International Space Station [Park et al. (2017)]. Other works utilised the *Payload Attachment Fixture* (PAF, also known as a Marman ring) of a target spacecraft as a grasping point. The PAF is ring adapter on the spacecraft, and it is used for mounting on the launch vehicle. Yoshida et al. (2004) initially used grapples to capture a target from its PAF. It was also the target for the planned E-deorbit mission by the European Space Agency [Wieser et al. (2015)], in which a clamp-like gripper would capture the PAF of the ENVISAT satellite. Similarly, Ratti (2015) presented a Launch Adapter Ring Capture Tool that uses laser positioning to capture a PAF. Hirano et al. (2017) proposed a retractable gripper design with two V-shaped end-effectors to implement a caging grasp on a PAF. A complete study of all phases of deorbiting a spent rocket stage by capturing the PAF was given by Felicetti et al. (2016).

Another component that has been used as a contact point is the bell of a spacecraft engine. It serves as a good point for capturing and deorbiting *Apogee Kick Motors* (AKMs), the upper stage of a rocket used for placing a payload in its final geostationary orbit. Yoshida and Nakanishi (2003) presented an impedance matching control method for inserting a probe in the AKM nozzle for minimum-disturbance capturing. A similar approach was followed by DLR for the proposed Experimental Servicing Satellite mission, where an expanding probe with force sensors targeted an AKM for deorbiting [Hirzinger et al. (2004)]. Hays et al. (2004) presented a similar AKM capturing method, but instead of a probe, they used a flexible cable-like probe, that can be retracted after capturing. McCormick et al. (2018) conducted a feasibility study of a cubesat equipped with a robotic clamp, that could capture the engine nozzle of a large piece of space debris and change its orbit.

Another spacecraft part candidate for grasping would be the triangular beam that connects the solar panel to the satellite body. Xu et al. (2010) proposed a method for detecting this triangular section with image processing methods and grasping it with a 3-fingered hand.

The aforementioned works propose the capturing of different parts of a payload in order to *mechanically restrain* it. To do so they usually require specialised grippers, designed to capture only one particular part of the spacecraft, with limited capability of generalisation to new parts. These robotic systems also show limited intelligence, as their design philosophy is that they only need to detect the satellite part and move to execute the mechanical grappling. By using simple 2-fingered grippers or multifingered hands and intelligent grasping algorithms, terrestrial robots are able to efficiently grasp objects of various shapes, analyse the grasp mechanics and leverage visual and other information of the object. In the case of multifingered hands, terrestrial robots can make grasp synthesis more efficient, by finding finger contact points that minimise well-defined grasp criteria. And while significant number of robotic hands for orbital applications exist [Biagiotti et al. (2001); Akin et al. (2002); Rubinger et al. (2002); Chalon et al. (2011); Bridgwater et al. (2012); Chalon et al. (2015); Zhao et al. (2016)], very few studies demonstrate intelligent, dexterous grasping applied in orbital robotics. Farrell et al. (2017) demonstrated dexterous *affordance-based grasping* with the NASA Robonaut2, where the robot gets a 3D point cloud of a scene, and a human user matches certain parts of the point cloud to a catalogue of known objects (handles, tools, buttons ea.). The robot then exploits learned object affordances (i.e., connections between objects, associated actions with the object, and hand configurations e.g., “button”-“push”-“extended index finger”) to execute the user-defined task in a shared-control architecture. Unsupervised learning has also been employed to synthesise grasping points on a spacecraft engine from a 3D point cloud [Mavrakis and Gao (2019)]. The robot autonomously selects the grasp that optimises predefined reachability criteria. Zhang et al. (2019) proposed a method for capturing orbital targets by caging them with a specialised end-effector. Despite using a bespoke end-effector, they were able to demonstrate that it can capture objects of various shapes.

A very important property in robotic grasping is *asymptotic grasp stability* i.e., the ability of the fingers resting on an object to return to the initial contact point in the presence of external disturbances. It is the property that enables terrestrial robots to hold objects rigidly. There is a vast existing literature of terrestrial robotics that deals with grasp stability, from classical works that investigate the problem of geometric analysis of a grasp to determine its stability [e.g., Montana (1991); Howard and Kumar (1996); Bruyninckx et al. (1998)], as well as synthesising new grasps from geometry data [e.g., Sahbani et al. (2012)] and learning grasp stability properties [e.g., Dang and Allen (2012)]. Generating stable grasps on orbital targets has essentially the same effect as *mechanically restricting* an object, similarly to most orbital-capturing methods mentioned above. A special case that achieves the effect of grasp stability, without elaborate grasp synthesis is presented by Jiang et al. (2017). The authors introduced a bio-inspired gripper that is able to stick onto a free-flying object and maintain rigid contact through adhesive pads. This method can achieve a strong grip, but the hit-and-stick grasping method severely limits the dexterity of the grasp.

In this paper we introduce a method for analysing the grasp stability on an orbital target. We aim to overcome the limitations of the works above by:

 1. Analysing the grasp stability on an orbital target, and determining whether a stable grasp can substitute mechanical locking.
 2. Performing stability analysis with existing methodologies from terrestrial robotics, that can be extrapolated to any orbital robot-target contact, and enabling dexterous planning.
 3. Utilising a simple hardware (2-fingered commercial gripper and 6-DOF robot arm) for the analysis, instead of equipment such as clamps, probes, interfaces, ea. designed particularly for the target.
 4. Proving the feasibility of our method thorough simulations and real-robot experiments.

**FIGURE 1:**
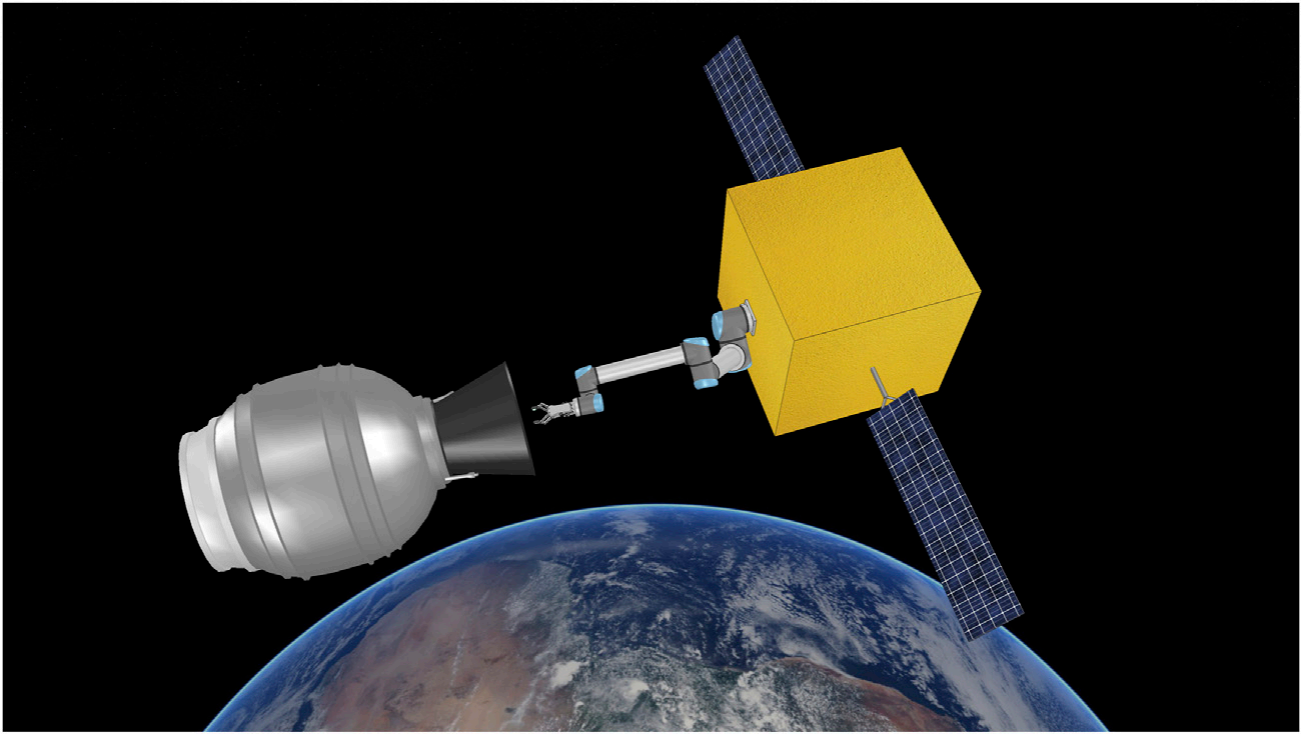
Simulation scene of a chasing spacecraft and a spent STAR-48 rocket stage. In this paper we evaluate how a robotic gripper could be used to capture space debris by analysing the grasp stability of the gripper-nozzle bell grasp.

We select the surface of an AKM engine bell to base our analysis, because it is a good candidate contact point, widely used in other studies. The structural robustness and curved surface increase the grasp stability. In our last paper [Mavrakis and Gao (2020)] we developed the theory for the stability analysis, a numerical evaluation, and preliminary simulation results. This paper extends the previous work by introducing more rigorous simulations with various targets, grasps and physics engines, as well as evaluating the methodology on a robotic testbed that can simulate microgravity conditions. We aim to provide an alternative way of capturing orbital targets efficiently and demonstrate the first application of classical robotic grasping to orbital robotics. An example scene of AKM capturing is seen on Figure 1.

## 2 Methodology

For context, we repeat the methodology of our previous work [Mavrakis and Gao (2020)], as well as the work of Howard and Kumar (1996) and Bruyninckx et al. (1998) on grasp stability analysis, on which we base our stability criterion.

### 2.1 Surface Curvature

Let $S_{A}$ be the surface of a robot finger, and $S_{B}$ a surface patch of the engine nozzle. Let also $O_{A}$ and $O_{B}$ be a coordinate frame on each surface with the *x* and *y* axes of each frame overlapping with the *principal curvature directions* of each surface. The *z* axis is orthogonal to the two other axes. The *z*-axes of frames of each surface are considered colinear. However, the orientation of *z*-axes around this colinear common normal can change by an angle of *ψ*. The principal curvatures of the two surfaces is noted with ($k_{a1}$, $k_{a2}$) and ($k_{b1}$, $k_{b2}$). The corresponding curvature matrices are noted as follows:

$$L_{A}=\left(\begin{array}{cc} -k_{a1} & 0\\ 0 & -k_{a2} \end{array}\right)$$ (1)

$$L_{B}=\left(\begin{array}{cc} cos\psi & sin\psi \\ sin\psi & -cos\psi \end{array}\right)\left(\begin{array}{cc} -k_{b1} & 0\\ 0 & -k_{b2} \end{array}\right)\left(\begin{array}{cc} cos\psi & sin\psi \\ sin\psi & -cos\psi \end{array}\right)$$ (2)

We model the robot finger pads as spheres. The spheres have radii $r_{f1}$ and $r_{f2}$, but for simplicity we assume $r_{f1}=r_{f1}=r_{f}$ for the rest of the paper. For each finger, we have $k_{a_{1}}=k_{a_{2}}=1r_{f}$.

**FIGURE 2:**
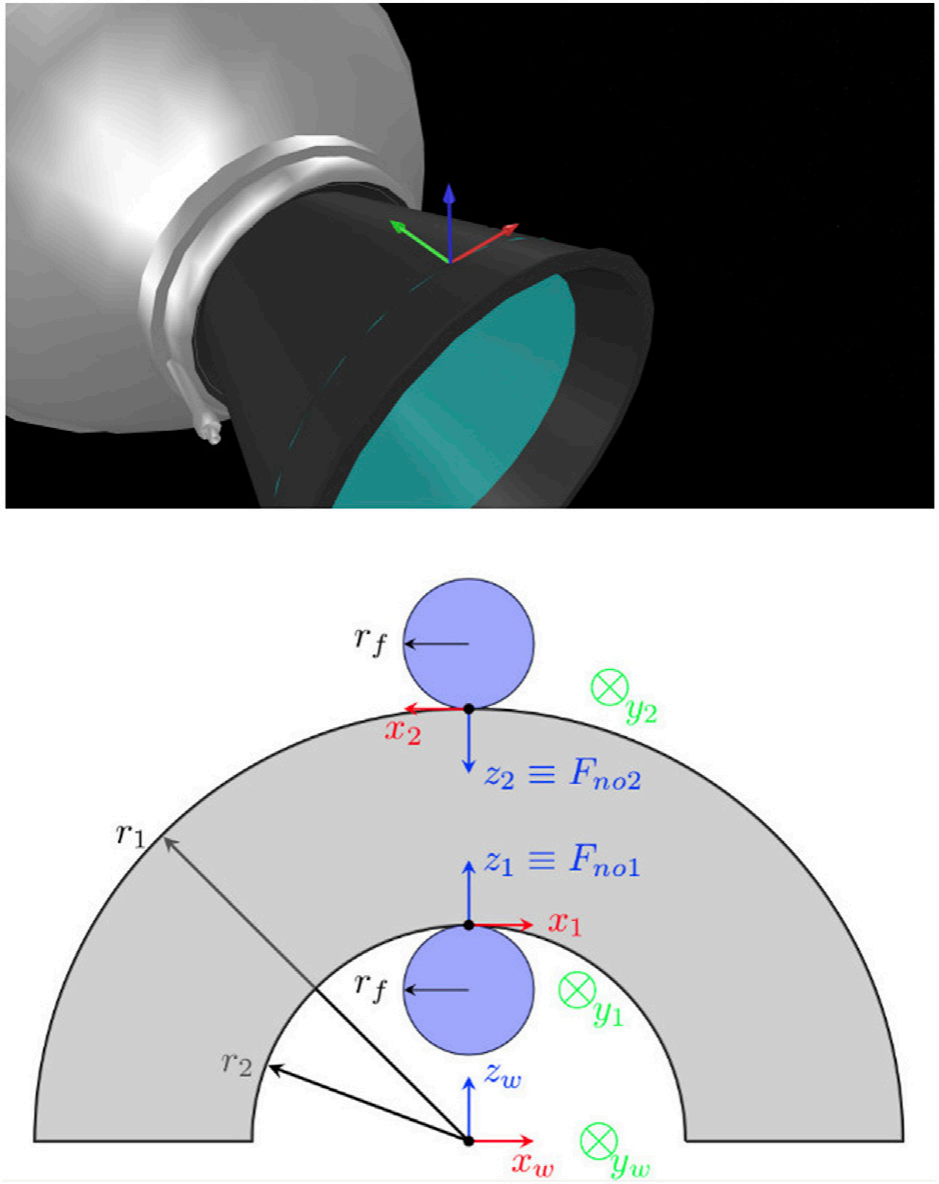
Top: a grasping pose on the bell surface, indicated with a coordinate frame. The green and red arrows show the direction of minimum and maximum curvatures (*y*-axis and *x*-axis). Bottom: frontal view of the AKM nozzle bell and robotic fingers, as would be seen by the robot’s camera. The coordinate frames on the fingertips are the frames $O_{A1}$ and $O_{A2}$. The respective $O_{B}$ frames are the principal curvature frames on the nozzle surface. For visualisation, we note the internal and external radii of the nozzle with $r_{2}$ and $r_{1}$, but for a real nozzle we have $r_{1}\approx r_{2}$. The normal force applied from each fingertip is $F_{n1}$ and $F_{n2}$. The grasp is *in equilibrium* if $F_{n1}=F_{n2}$.

The engine nozzle has a roughly conic shape, with one direction appearing almost planar, and the perpendicular direction curved with a variable radius of *r* (shown with blue disk radius in Figure 2). The principal curvatures of a point on the nozzle surface are $k_{b1}=\frac{1}{r}$ (red axis of Figure 2), $k_{b2}=0$ (green axis of Figure 2). By changing the values of *ψ*, $r_{f}$ and *r*, we can analyse the grasp stability of various finger sizes on different areas of the engine cone, under pivoting of the finger on the underlying surface.

### 2.2 Intrinsic Stiffness Matrix in Weightlessness

Howard and Kumar (1996) were the first to introduce the *Intrinsic Stiffness Matrix* (ISM) of a grasp as a measure of its stability. The ISM is affected by the positioning of the fingers on an object w.r.t. a reference frame, the finger applied forces, the combined local curvatures of both finger and object, and frictional and other material properties of the contact. The fingers that support the contact points are assumed to be non-compliant. When this is not the case, the *Grasp Stiffness Matrix* needs to be used, stability analysis that encodes both the finger compliance and grasp geometry. We assume rigid fingers in this paper. We do this to develop a more transferable stability criterion that depends more on the finger positioning on the object and ignores the effect of the finger compliance and structure, which vary along different robotic grippers and hands. The stability is determined purely by the ISM in this case.

Given a set of placed fingers on an object’s surface (i.e., a grasp), the ISM relates the variation of the total applied wrench on the object from all fingers, to the variation of the total object displacement incurred by this wrench. This is the second-order variation of the grasp’s potential energy:

$$\text{Δ}\vec{F}=-K_{o}\text{Δ}\vec{x_{o}}$$ (3)

where $K_{o}$ is the 6×6 ISM, $\vec{F}\in R^{6}$ is the total applied wrench that all contacts apply on the object, and $\vec{x_{o}}\in R^{6}$ is the object translation and rotation vectors, which we express on a global reference frame $O_{w}$. $K_{o}$ is symmetric when the grasp is in force-equilibrium i.e., the sum of all the applied forces by the fingers does not move the object and resist all possible external forces.

$$Kc=\left(\begin{array}{cccc} F_{no}L_{A}{\left(L_{A}+L_{B}\right)}^{-1}L_{B}+k_{t}I_{2\times 1} & 0_{2\times 1} & -F_{no}L_{A}{\left(L_{A}+L_{B}\right)}^{-1}\text{Λ} & 0_{2\times 1}\\ 0_{1\times 2} & k_{n} & 0_{1\times 2} & 0\\ \left(M_{o}L_{A}-F_{no}\right)\text{Λ}{\left(L_{A}+L_{B}\right)}^{-1}L_{B} & \text{Λ}F_{to} & \left(F_{no}\text{Λ}-M_{o}L_{A}\right){\left(L_{A}+L_{B}\right)}^{-1}\text{Λ} & 0_{2\times 1}\\ F_{to}^{T}\text{Λ} & 0 & 0_{1\times 2} & k_{\theta } \end{array}\right)$$ (4)

Eq. 3 is also applicable for each contact and relates the variation of a single finger-applied wrench to the total object displacement. In the single-contact case, we define the *contact stiffness matrix* $K_{c}$ as a function of the combined finger-surface local curvature, the applied force by the finger, and the frictional and material properties of the contact. $K_{c}$ is given in Eq. 4. $k_{t}$, $k_{\theta }$ and $k_{n}$ are the shear, torsional, and compressive stiffness constants respectively. They are functions of the fingertip’s shear and elastic moduli, and the contact cross-section dimensions [Cutkosky and Wright (1986)]. $M_{o}$ is the torsional finger moment. $F_{to}$ are the tangential frictional forces that exist on each contact. In order to prevent contact sliding, the tangential forces need to be within the *friction cone* of the contact. We also define, $\text{Λ}=\left(\begin{array}{cc} 0 & 1\\ -1 & 0 \end{array}\right)$ Each finger applies a normal force $F_{no}$ on the surface. After calculating the contact stiffness matrix $K_{c_{i}}$ for each contact, we combined them to construct ISM as follows:

$$K_{o}=\overset{N}{\underset{i=1}{\sum }}T_{i}^{T}K_{c_{i}}T_{i}+T_{mg}^{T}K_{c_{mg}}T_{mg}$$ (5)

where *N* is the total number of contacts on the object, $T_{i}$ is a matrix that transforms the contact coordinate frame $O_{i}$ to the global reference frame $O_{w}$ and $T_{mg}$ transforms the object’s centre of mass to $O_{w}$. $K_{c_{mg}}$ is an equivalent stiffness matrix for the gravity force. A variation in the object’s motion does not alter the gravity force, but instead, it moves the spatial coordinate frame of the object’s centre of mass, resulting in a change of the applied torque from the finger contacts. $K_{c_{mg}}$ is a stiffness matrix that connects this applied finger torque variation to the object displacement. In space, we have weightlessness, and this manifests by assuming $g=0\;m/s^{2}$. This means that the object’s weight does not affect the intrinsic stiffness of the grasp, which results in $K_{c_{mg}}=0_{6\times 6}$, and Eq. 5 becoming:

$$K_{o}=\overset{N}{\underset{i=1}{\sum }}T_{i}^{T}K_{c_{i}}T_{i}$$ (6)

Eq. 6 means that the grasp stability in a weightless environment is a function of the grasp geometry, force, and material properties, and not of the object’s weight.

### 2.3 Grasp Stability Criterion

Under non-compliant fingers, the stability of the grasp is determined by the eigenvalues of the ISM. If $K_{o}$ is positive-definite, the eigenvalues of $K_{o}$ are all positive, and the grasp is *asymptotically stable*. If at least one eigenvalue is negative the grasp is *unstable*, and if one or more eigenvalues are zero (and all others positive) the grasp is *marginally stable*. Higher-order contact motion analysis is required to determine the stability.

As mentioned, a global reference frame $O_{w}$ is required for the calculation of $K_{o}$. As described by Bruyninckx et al. (1998), the *value* of the eigenvalues of $K_{o}$ is not invariant to changes of $O_{w}$, but their *sign* remains the same. We can use the sign if the eigenvalues to determine grasp stability, however if we wish to change $O_{w}$, the analysis must be repeated, and the results can not be used to determine which choice yields stabler grasp. Bruyninckx et al. (1998) developed a stability measure based on the generalised eigendecomposition of $K_{o}$, and a 6×6 matrix *M*. They derive the matrix $M^{-1}K_{o}$ and they state that it is positive definite if and only if *M* and $K_{o}$ both are positive definite. *M* can be arbitrary but Bruyninckx et al. (1998) proposed the use of the grasped object’s mass matrix as a metric with *kinetic energy* information. When both $K_{o}$ and the matrix *M* are expressed in $O_{w}$, the eigenvalues of $M^{-1}K_{o}$ are invariant to the location of $O_{w}$. The eigenvalues of $M^{-1}K_{o}$ can then be used for qualitative stability analysis and grasp comparison. As a stability measure, we must examine the *minimum* eigenvalues of multiple grasps on a target, and determine the stability based on the larger minimum eigenvalue. If the mass matrix is used as the *M* matrix one can determine the stability of a single grasp on objects with same geometry but different mass distribution (i.e., one $K_{o}$ and multiple *M*).

We use the above analysis for analysing the stability of a robotic grasp on the surface of an engine’s nozzle. We build $K_{o}$ from a 2-fingered gripper grasping the engine cone (Figure 2). We then use the mass matrix of a target and the minimum-eigenvalue stability criterion. In reality, the mass matrix of an orbital target may not be known, but we test with a range of mass matrices to assess the proposed methodology’s stability limits.

### 2.4 Effect of Grasping Parameters on Stability

Each of the grasping parameter affects the minimum eigenvalue of the $M^{-1}K_{o}$ criterion. In our previous work Mavrakis and Gao (2020) we offered an initial study of how stability is affected under variations of only one parameter, in a capturing scenario where all other parameters are changed. Assuming the grasp pictured on Figure 2, we changed the following parameters one at a time: finger radius $r_{f}$, nozzle radius *r*, fingertip angle on the surface *ψ*, applied force $F_{n}$, and target mass *m*. We observed how the minimum eigenvalue of $M^{-1}K_{o}$ varies under alterations of each parameter. The results indicated that high grasping forces, lower target mass, and a ratio $\frac{r_{f}}{r}\to 1^{-}$ tend to increase the grasp stability. The outer and inner nozzle values ($r_{1}$ and $r_{2}$ in Figure 2) are typically almost equal to each other and equal to *r*, and we found that their difference does not affect stability after some first numerical tests. We utilise these results to select design values for the experiments presented in this paper.

## 3 Physical Testbed Setup

The physical experiments in this research are carried out on the novel orbital robotic testbed at STAR LAB, Surrey Space Centre. There are two main types of orbital robotic testbeds to off-load gravity and simulate the dynamics of the experimenting target in the micro-gravity environments-using air-bearing tables and using robotic arms. The air-bearing tables offer 3-DOF real motion [Virgili-Llop and Romano (2019)], while the robotic arms can deliver up to 6-DOF full dynamic motion with the cost of dynamics fidelity and increased system complexity [Artigas et al. (2015); Colmenarejo et al. (2018)]. The STAR LAB’s testbed belongs to the second category [Hao et al. (2019)].

Three key aspects of the orbital robotic testbed are:

 1. The setup is re-configurable, which means it can use various grippers and sensors.
 2. The testbed is fully integrated with the Robot Operating System (ROS), which means it accesses various packages from the wider range of robotic community and industrial support to boost its versatility.
 3. The testbeds offers different fidelity modes for orbital dynamics and mechanics simulation, which is suitable for diverse orbital robotic experiments.

**FIGURE 3:**
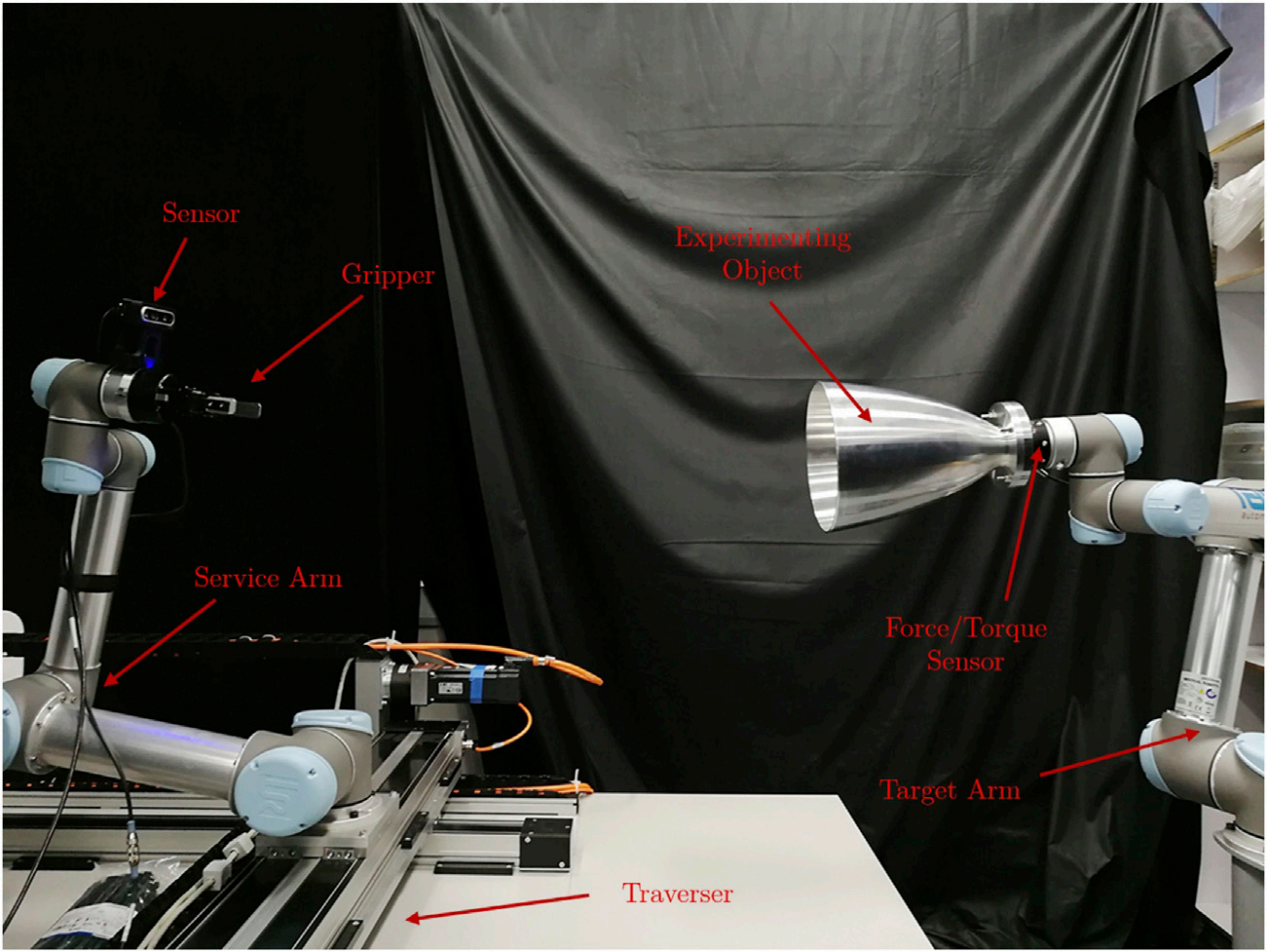
The nominal configuration of the STAR LAB’s orbital robotic testbed for the experiment of grasping of a spend rocket stage. The sensor is Intel RealSense D435. The gripper is RobotiQ 2F-85. The force torque sensor is RobotiQ FT300.

The nominal setup of this re-configurable testbed comprises of two collaborative UR5 robot arms-the service arm and the target arm, an in-hand sensor, a gripper, and a customised 2D traverser to simulate the service spacecraft, as seen in Figure 3. The service arm sits on top of the traverser while the target arm carries the experimenting target. The sensor and gripper can be selected according to various experiments. The whole setup fades into a lighting-controlled vicinity covered by the light-absorbing 45% polyacrylic +55% cotton fabric and supplied with Halogen light sources to recreate the orbiting lighting condition.

A real-time dynamic responsive approach is used to simulate the experimenting object dynamics as free-floating in the micro-gravity environment. In this setup, the interacting forces of the gripper acting on the target module are gathered by the force-torque sensor on the target arm. Two methods developed to utilise the forces/torques-direct dynamics simulation and relative position simulation. The relative position simulation method exploits the forces/torques information to directly update the relative motion of the target using, for instance, the Clohessy-Wiltshire equations, then the target arm tracks the relative trajectory and attitude of the target as in free-floating condition.

**FIGURE 4:**
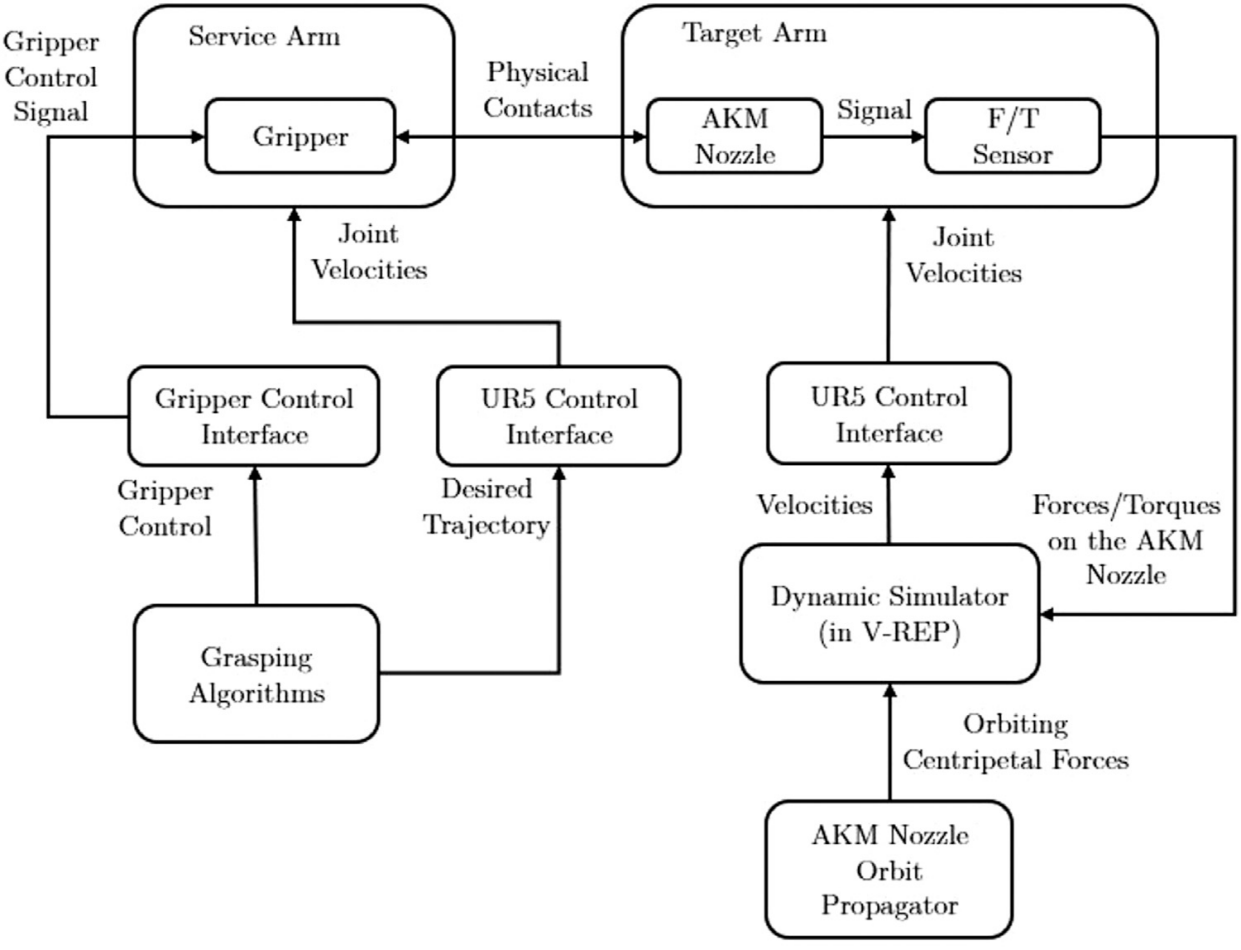
The flowchart of the STAR LAB’s Space Robotics Testbed with the configuration for the AKM grasping and pulling hardware-in-the-loop experiment.

In this research, we use the direct dynamic simulation method. The direct dynamics simulation, whose flow chart is shown in Figure 4, exploits the forces/torques and then applies them to an equivalent model in a selected dynamic engine, integrated with the V-REP simulator [Rohmer et al. (2013)]. From there it directly calculates the target twist to be applied on the experimenting object. Since each of the systems communicate within the ROS framework, the simulated velocities are then sent to the UR5 control interface to command the robot arm to follow the velocities. Furthermore, the direct simulation method has two different modes-high-fidelity mode and post-capture mode. The high-fidelity mode, in addition, updates the orbital dynamics hence the experimenting object is always experiencing a centrifugal force towards a user-defined Earth Centrals Inertia (ECI) Point. In this case, the ECI origin will be set as the nominal flying altitude of the AKM. While in post-capture mode, the orbital dynamics are less relevant because we assume a well-established link between the AKM and the gripper, therefore null relative motions between them. In our physical testbed experiment, only the post-capture mode is used as the experiment focuses more on the grasping and pulling other than the high-fidelity relative navigation.

Once more, the orbital robotic testbed is re-configurable, the special hardware and their specifications used in this research are covered in Section 4.2 and introduced along with the experiment.

## 4 Experimental Results

We set three types of experiments to verify the stability of a robotic grasp on the bell of an AKM, and check how different parameters affect the grasp success or failure. All experiments consist of a chaser spacecraft capturing an AKM and pulling it towards the chaser body, and seek to evaluate the stability of the grasp in terms of the detected slippage and grasp outcome when a number of experimental parameters vary.

### 4.1 Simulation

The first experiment is performed on the V-REP simulator. We set up a simulation scene where objects are weightless. The gravity acceleration was set to $g=-10^{-7}\frac{m}{s^{2}}$, as the simulator produces errors if *g* is set to zero.

The simulation scene consists of a chasing spacecraft with a mass of 500kg, equipped with a 6-DOF robotic manipulator and 2-fingered gripper. The manipulator is a Universal Robots UR10, but we have increased the V-REP model’s joint torque limits. Similarly, the 2-fingered gripper is modelled after an OnRobot RG2 gripper, with increased joint torque in order to amplify the applied force. We also augment the gripper fingers with spherical pads, to consist with the analysis of Section 2.1. The pads have a radius of $r_{f}=0.01m$, a value close to typical gripper fingerpad dimensions. The friction coefficient of the contact between finger pads and bell surface was set to μ=1.6, to resemble an aluminium-to-aluminium dry contact in vacuum [Deulin et al. (2010)]. Aluminium is a widely used material in space applications, although in reality the coefficient would be affected by other parameters such as temperature, propellant residue after burning ea.

**FIGURE 5:**
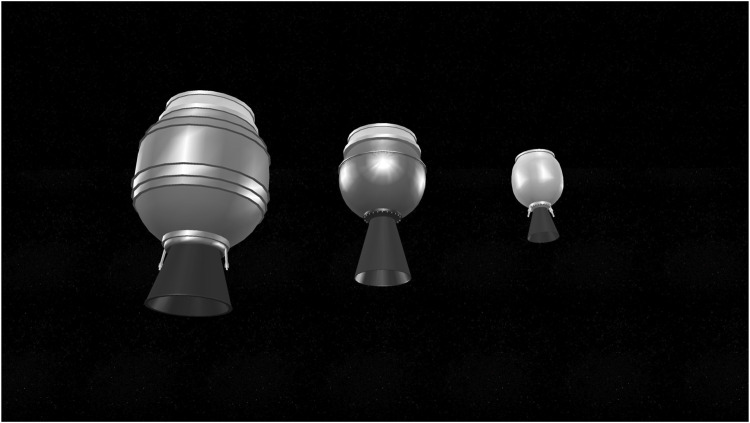
The three upper stages models used in the V-REP simulation. From left to-right: STAR-48b, STAR-24c, and STAR-13b, all manufactured by Orbital ATK. The models have been designed according to the dimensions and mass parameters provided by the manufacturer [Orbital ATK Inc. (2018)].

As the mass and mass distribution are some of the multiple parameters that affect grasp stability, we perform our experiments with three different AKM targets of varying inertial parameters. The targets are modelled after existing motors of the STAR family, manufactured by Orbital ATK (formerly Thiokol). Such motors have been used in launches with numerous launch vehicles, including the Space Shuttle. The reasons for selecting these motors is their easy-to-model shape and relatively small dimensions and mass, compared to other frequently used upper stages. The selected motors present a realistic test case, as they have been used for launches for more than 50 years, and they tend to stay in orbit from weeks to decades. The dimensions and masses for the simulated targets were set in accordance to the manufacturer’s specifications [Orbital ATK Inc. (2018)]. The simulated targets are shown in Figure 5. They are modelled after the STAR-48b, STAR-24c and STAR-13b motors, and were chosen to correspond to a heavier (STAR-48b, dry mass 131 kg), a moderate (STAR-24c, dry mass 19.7 kg) and a lighter target (STAR-13b, dry mass 5.8 kg). In the absence of available mass distribution data from the manufacturer, the total mass distribution was calculated by calculating the mass distribution of empty spherical (or near spherical) shells, and combining them with cylindrical mass distribution of the conical nozzles. The materials to calculate the density for each part were extracted from the manufacturer’s specifications [Orbital ATK Inc. (2018)].

The main task for the robotic arm is simple: to capture the AKM nozzle bell at a defined grasping point, and pull back the target over a total pulling distance of 0.3m. Such pulling distance is typical for proximity operations of spacecraft, where the target and the chaser can be at a distance lower than 1m. It is also a distance that suits our testbed experiment (explained in the next Section), since it represents a sufficient pulling distance to evaluate stability but it does not bring our robots to a singular position due to excessive stretch. We measure the slippage of the robotic fingers on the bell surface during the pulling motion. If the grasp retains hold of the target when the pulling motion ends the grasp is considered successful, and if the target slips from the fingers at any point during the pulling motion, the grasp fails. The purpose of the simulation is to evaluate the grasp success under variation of the grasping location on the target and pulling velocity. We keep the grasping force constant along all grasps, equal to 180 N. The robot reaches each grasping point through an approaching pose of same orientation and translational offset of 0.3m. The gripper and robotic arm are position-controlled for the whole experiment, and their compliance does not change during the capturing or pulling motion. In addition, the spacecraft base attitude is not controlled, i.e., the base is free-floating for the grasping and pulling motion. This would not be the case in reality, as the capturing of the target needs to be conducted with arm impedance controllers and rigidisation algorithms that ensure minimal disturbance caused to and by the target. We choose to leave the arm and gripper non-compliant, and the base uncontrolled to artificially introduce additional disturbances on the grasp, and determine its stability under “worst case” conditions.

**FIGURE 6:**
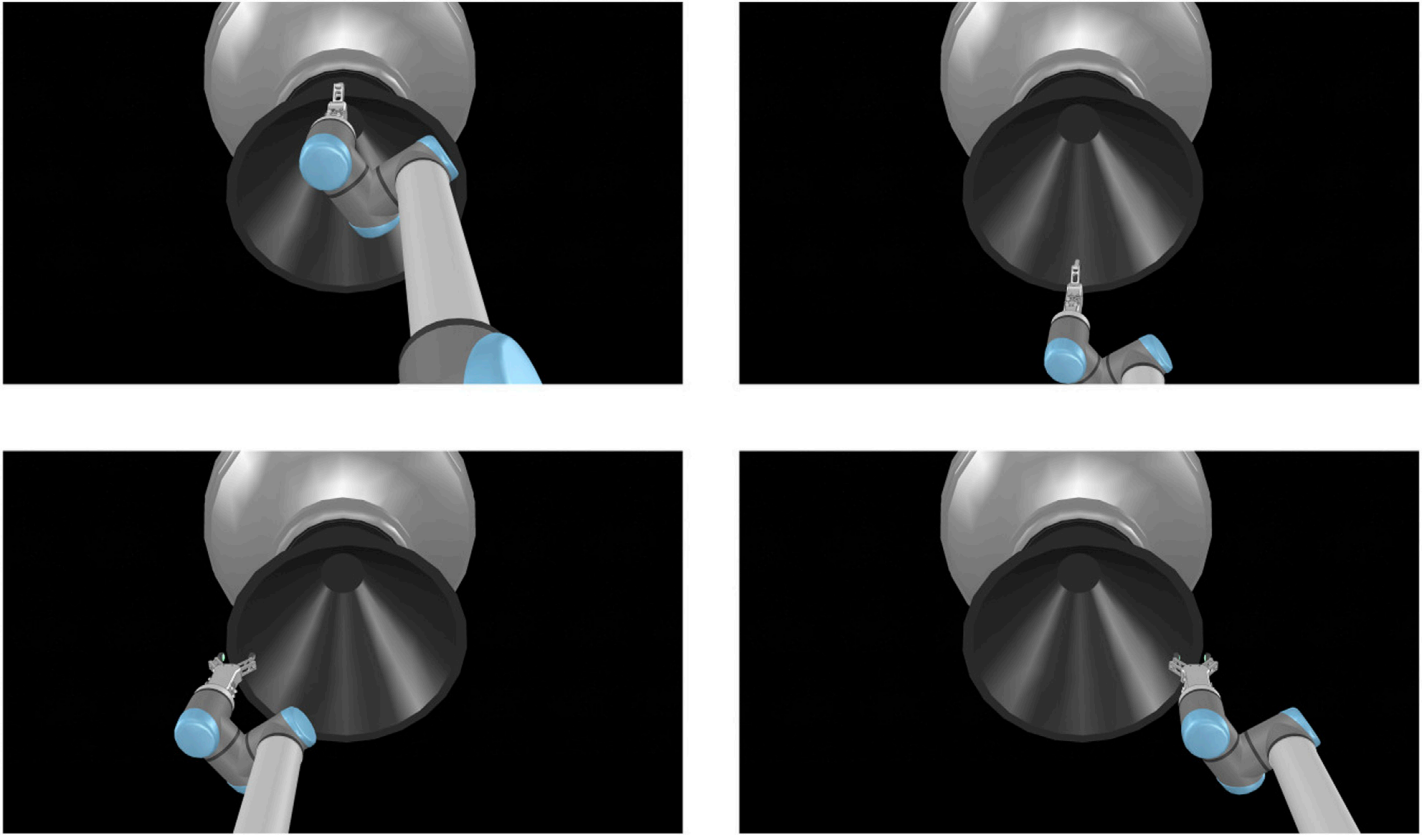
The four grasping points for each target of the simulation, shown here for STAR-48c: Top grasp **(upper left image)**, bottom grasp **(upper right image)**, left grasp **(bottom left image)**, and right grasp **(bottom right image)**.

In our previous work [Mavrakis and Gao (2020)], we determined the stability of a single grasp on an AKM in a similar method using the Newton physics engine supported by V-REP, observing very low finger slippages. To ensure that the results are consistent with other physics engines, we now conduct our simulation using three of the supported engines of V-REP, namely Bullet 2.78, ODE and Newton. We select four grasping points on the surface of each AKM, namely “top”, “bottom”, “left”, and “right”, shown in Figure 6. The grasps were selected manually in the simulator, without employing any synthesis algorithm. They were selected to cover the whole circumference of the nozzle rim, and their stability was not calculated before the pulling experiment. Instead, the aim was to evaluate their experimental stability after the pull. We also define three pulling profiles for the robot end-effector to follow, with velocity values 0.02, 0.04, and $0.06\frac{m}{s}$ and constant acceleration of $0.01\frac{m}{s^{2}}$. The pulling profiles are relatively slow for terrestrial robotic applications, but are typical of the velocities used in orbital robotics.

For clarity, we distinguish between “grasp” as in grasping point location on the target surface, and “grasp” as in the *grappling motion* with given simulation parameters, we define an *attempt* as a simulation run that constitutes of a combination of grasping point, velocity, and physics engine as follows:

Attempt=[graspingpoint,velocity,engine] (7)

In total, we have four grasping points, under three motion profiles, and for three physics engines, i.e., 4*3*3 = 36 attempts.

**FIGURE 7:**
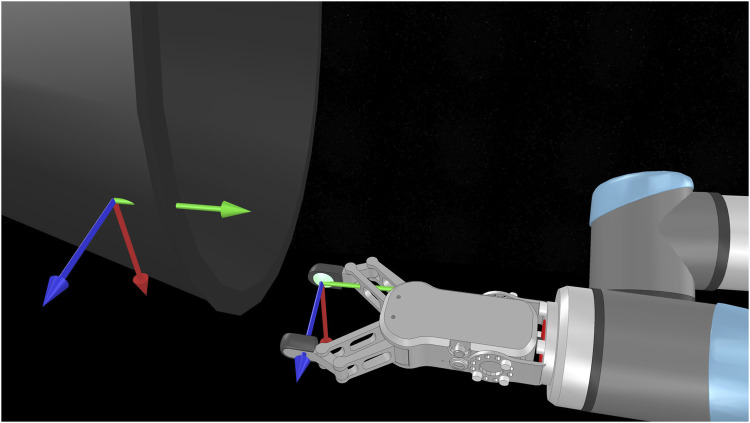
The grasping and right fingertip coordinate frames $S_{B2}$ and $S_{A2}$ (**left and right** frames on the picture). In V-REP, we can directly measure the distance of the fingertip frames w.r.t the grasp frame, and the rotation over the axis perpendicular to the surface. The distance and rotation constitute the measured slippages.

To measure the slippage, we superimpose a dummy point on the grasping point immediately after contact (Figure 7). The dummy point is essentially a coordinate frame with the same position and orientation as the local coordinate frames $S_{A1},S_{A2}$ of each fingers, that is part of the nozzle and moves with it. The slippage is calculated as the Euclidean planar distance ($d=\sqrt{dx^{2}+dy^{2}}$, with *x*,*y* directions parallel to the nozzle surface, red and green axes of 7) and pivot angle (rotation angle w.r.t. *z* axis, perpendicular to the nozzle surface, blue axis of 7) of the gripper pad on the nozzle surface. We take the measured slippages for each finger $d_{1},\;d\theta_{1}$ and $d_{2},\;d\theta_{2}$ for each finger, and average them to show the final slippage d, dθ. For an ideal, successful and rigid grasp, all values should be close to zero for the whole pulling duration.

**FIGURE 8:**
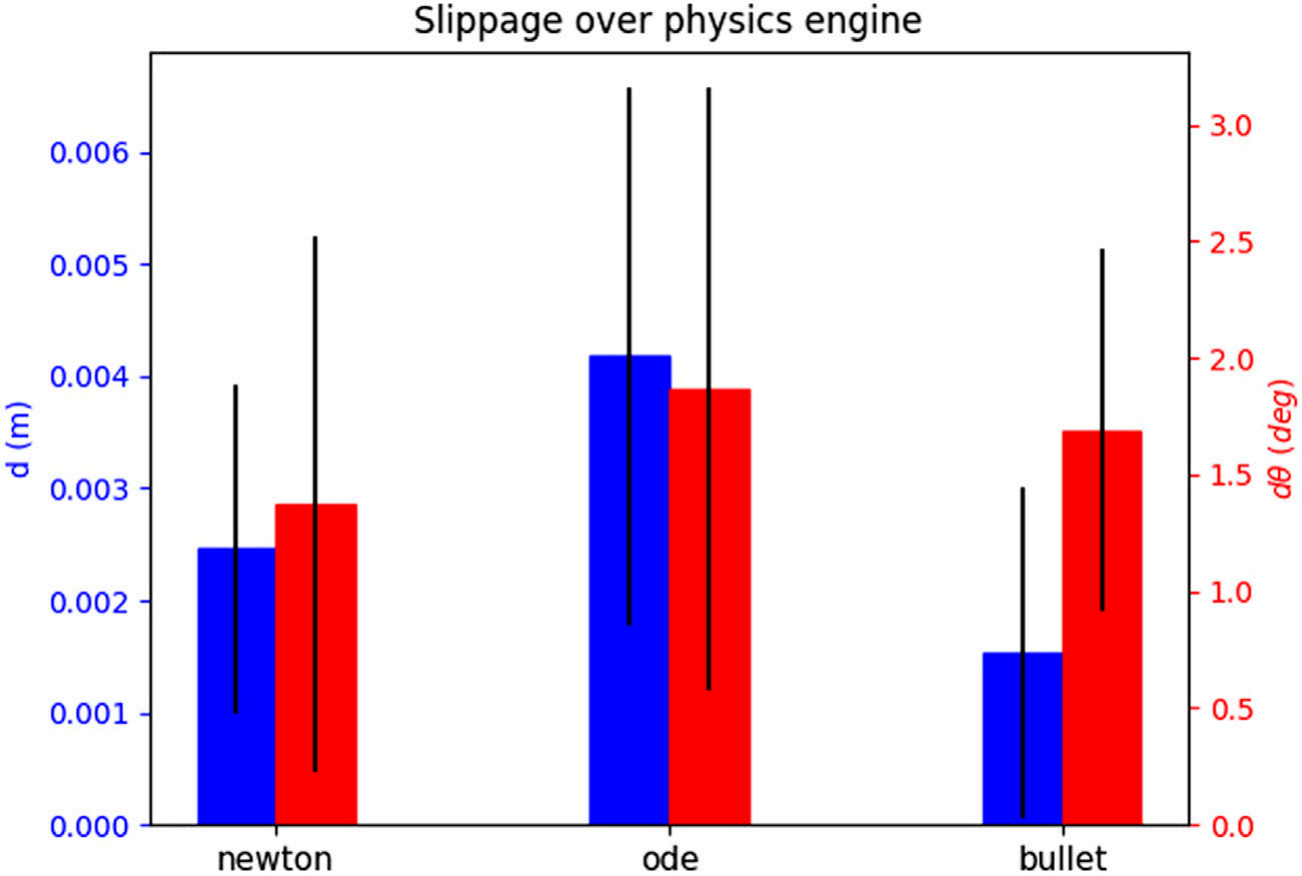
The effect of the physics engine on grasp stability for the simulation experiment. The blue bars show the mean and standard deviation of the translational slippage, and the red bars those of the rotational slippage.

**FIGURE 9:**
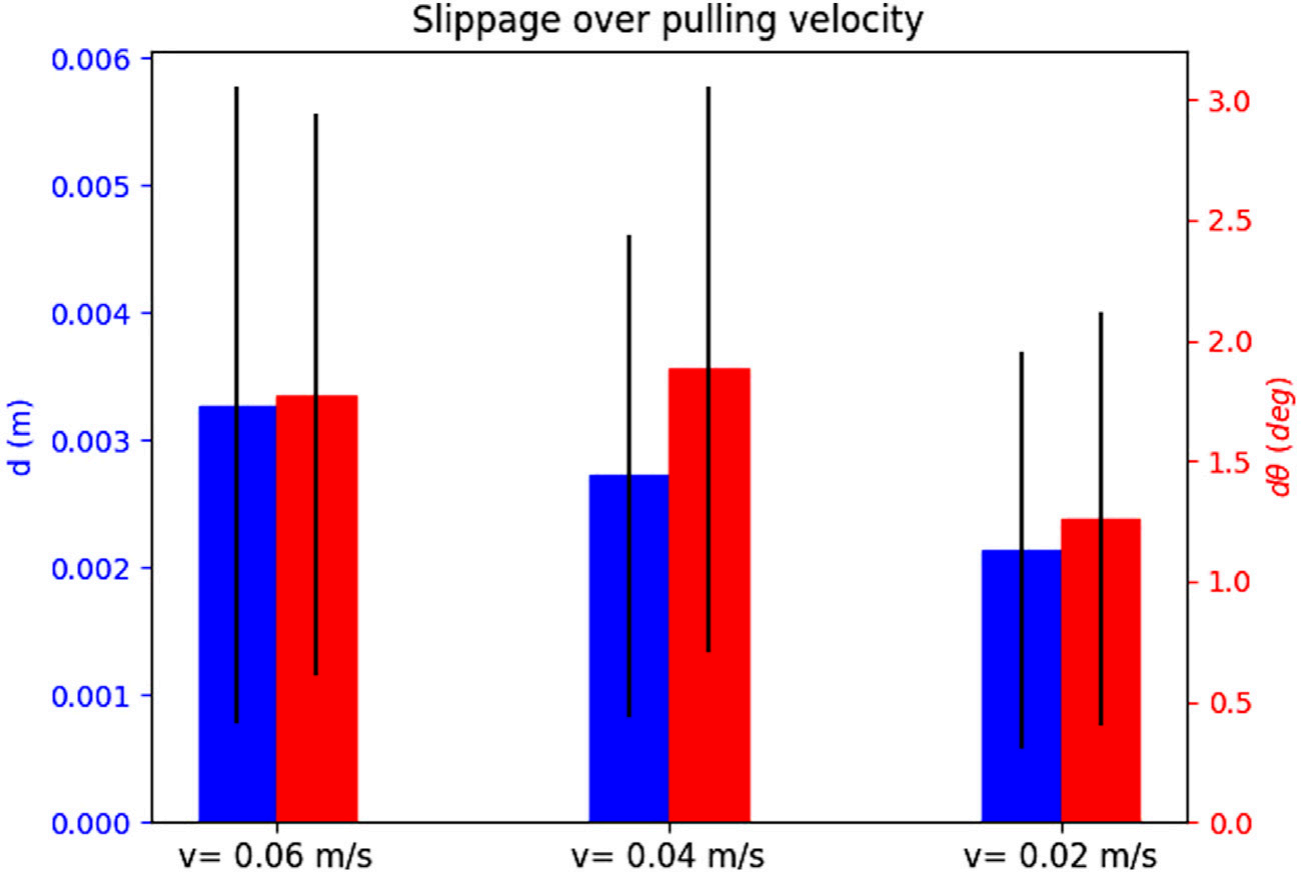
The effect of the pulling profile on grasp stability for the simulation experiment.

**FIGURE 10:**
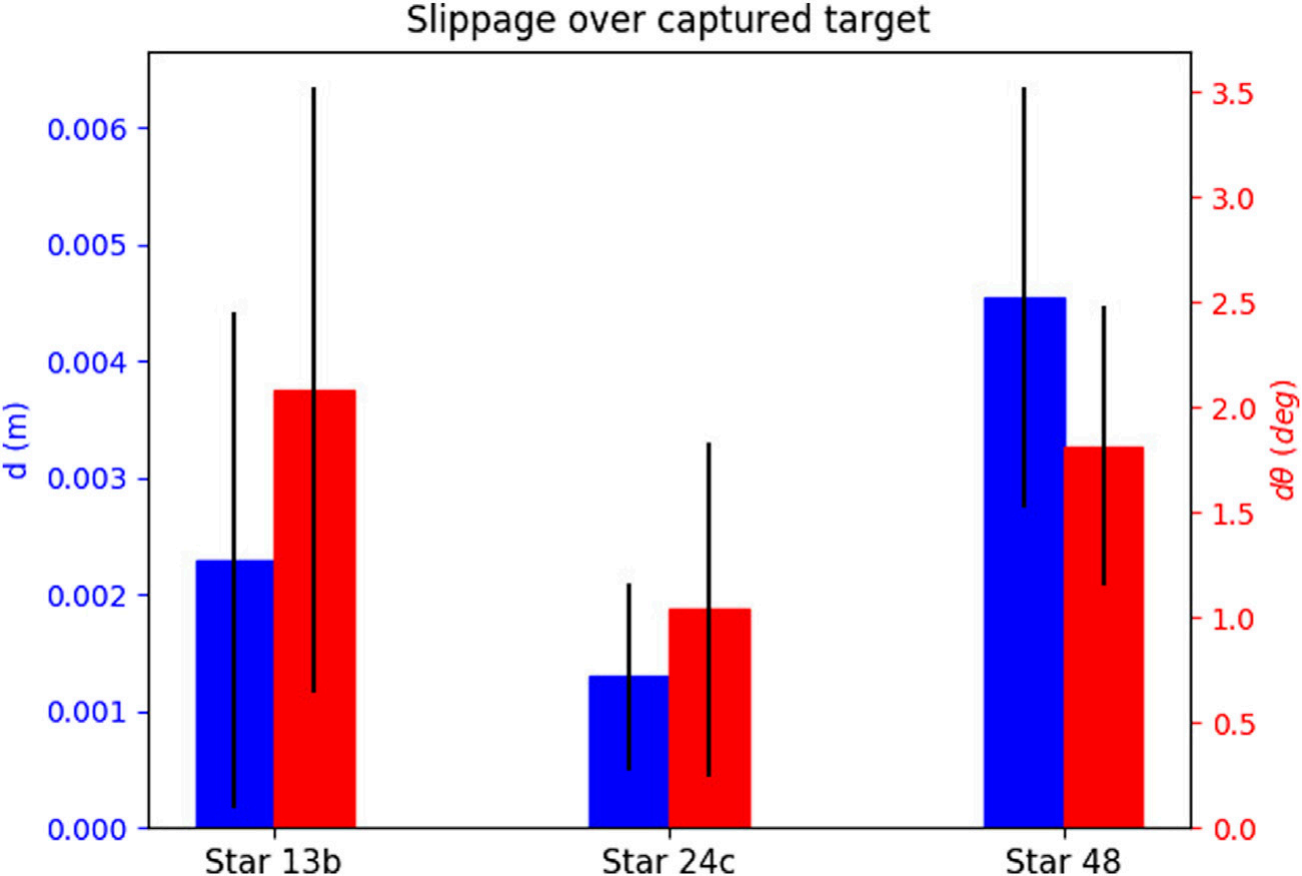
The effect of the selected target on grasp stability for the simulation experiment.

**FIGURE 11:**
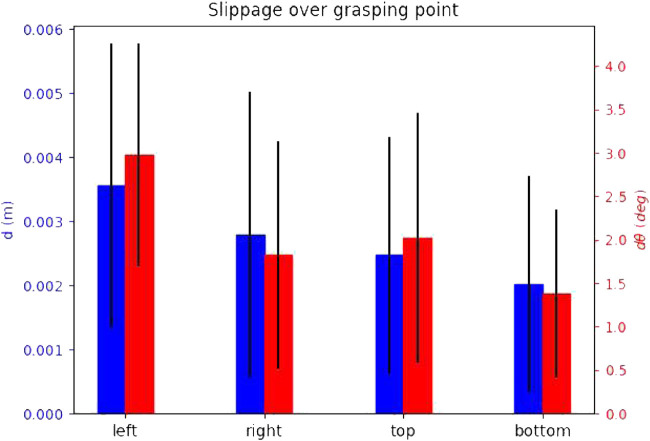
The effect of the grasping point on stability for the simulation experiment.

For each attempt, we measure the slippages as time signals, and note the maximum slippage values observed over the whole duration of the attempt (i.e., the maximum for each slippage signal). In order to showcase how the slippage is affected by each of the parameters that constitute an attempt, we select one attempt parameter and show the mean and standard deviation of all maximum values. We explain this for Figure 8 as an example. We wish to see how the choice of the physics engine affects *d* slippage. The physics engine parameter has three values, i.e., “Newton”, “ODE”, and “Bullet”. We look at the total set of maximum slippages for the 35 successful attempts, and we calculate the mean and standard deviation of all *d* maxima that are associated with each physics engine value (12 for Newton, 11 for ODE, and 12 for Bullet). The results are shown in Figure 8. We extend this process for all parameters that constitute an attempt. We show the effect of the physics engine, pulling profile, target, and grasping point in Figures 8–11 respectively. In total, only one attempt failed to hold the target for the whole pulling duration and 35 succeeded.

The results yield overall low slippages over all successful grasps. In many cases the slippage is in sum-centimetre magnitude, and the rotational slippage is also very low.

The physics engine seems to have little effect on the maximum slippage. The observed differences between translational slippages are a couple of millimetres at most, and for the rotational slippage less than $3^{o}$. These results validate the outcome of our preliminary work Mavrakis and Gao (2020), and shows that the low slippages are independent of the choice of the physics engine.

The slow pulling profile yields overall lower slippage values both for rotational and translational slippage. As the velocity increases, the slippages also become larger.

The attempts seem to yield very stable grasps for all targets. Finally, the slippage seems distributed over the grasping points, and no “best” grasping point can be decided. This outcome is intuitive, as the local contact geometry and pulling direction for all grasping points is the same.

### 4.2 Testbed Experiment

The second experiment was conducted on the testbed described in Section 3. We consider a similar scenario of chasing spacecraft in proximity to a target, that attempts a grasping and pulling motion. The traverser of the testbed is not used in the experiment, as emulating the approach phase is out of the paper scope. We mount a RobotiQ FT-300 force-torque sensor on the target arm for the real-time force measurement and orbital motion emulating. The gripper used is a RobotiQ 2F-85 model, with planar fingers, in the absence of rounded gripper pads. The planarity of the fingers on a curved surface would theoretically lead to a *marginally stable* grasp, as was shown in our previous work [Mavrakis and Gao (2020)], however it was expected that the real friction between the two surfaces would increase the stability, an assumption both intuitive and mathematically explained by Howard and Kumar (1996).

For emulating the AKM to be captured, we mounted a machined aluminium nozzle bell on the target. The nozzle bell has roughly the same dimensions as a STAR-13b nozzle, with an exit diameter of r=0.22m. For the target orbital motion emulation, the force read by the F/T sensor was applied to a simulated STAR-13b model on a weightless V-REP scene with the Newton physics engine. The force application produced a target twist, that was transformed to the end-effector of the UR5, and fed to the robot with a frequency of 125 Hz.

**FIGURE 12:**
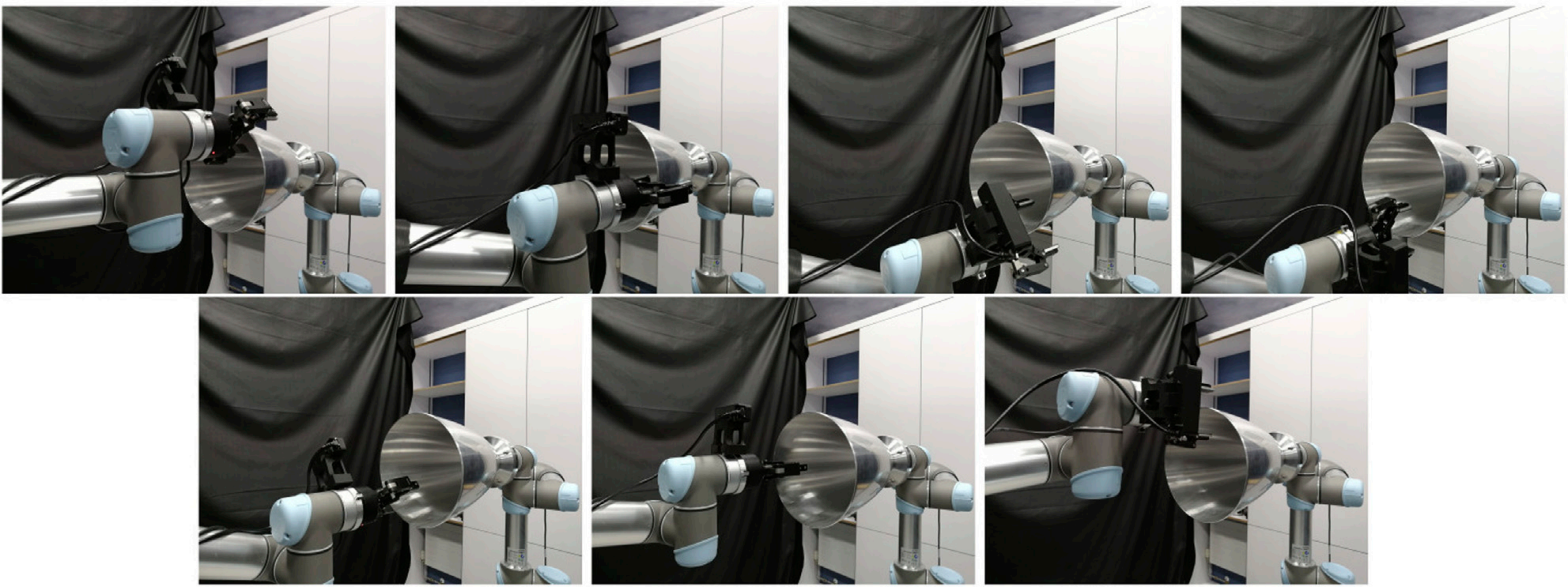
The seven grasps generated by manually placing the robot gripper on the nozzle.

**FIGURE 13:**
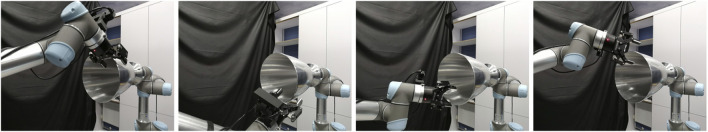
The four grasps generated by running the grasp synthesis algorithm of Mavrakis and Gao (2019).

We use the UR5 freedrive button to manually generate seven grasping points on the target nozzle, with roughly zero yaw angle of the gripper pads w.r.t. the nozzle rim (rotation w.r.t. to the finger contact *z*-axis). In addition, we used an Intel Realsense D435 depth sensor and the grasp synthesis algorithm presented in our recent work Mavrakis and Gao (2019) to extract four additional grasping points on the nozzle surface. The algorithm produces grasping points that have yaw angles within an adjustable specified range, which we set to $\left[-30^{o},+30^{o}\right]$. The seven manual and four generated grasps are shown in Figures 12, 13 respectively. The grasping force that the motor of the RobotiQ 2F-85 gripper should produce on each grasp was set to 150 N. As with the simulation experiment, each grasping point is reached through an approaching pose of same orientation, and translation offset of 0.3m.

We used three pulling profiles for the testbed experiment: one slow with $v=0.02\frac{m}{s}$ and $a=0.1\frac{m}{s^{2}}$, one moderate with $v=0.04\frac{m}{s}$ and $a=0.5\frac{m}{s^{2}}$, and one fast with $v=0.06\frac{m}{s}$ and $a=1\frac{m}{s^{2}}$. While the velocities match those of the simulation experiment, as well as realistic velocities that are used by robots on orbit, we had to gradually increase the acceleration values because when the UR5 robots were in contact in low accelerations, one of the arms perceived the persistent applied contact of the other as additional non-calculated payload, and entered a protective-stop mode halting the experiment. To minimise this effect, we used high accelerations, executed each capture a number of times, and kept the executions where the pulling motion was completed without interruption. In addition, the pulling distance was set to 0.25m, to be well within the common workspace of the two robots, avoiding singularities.

As we have one emulated target and no physics engine, in the real experiment, an attempt consists only of the pulling profile and grasping point, i.e., attempt=[profile,grasp]. We have 11 grasps and three pulling profiles, a total of 33 attempts.

**FIGURE 14:**
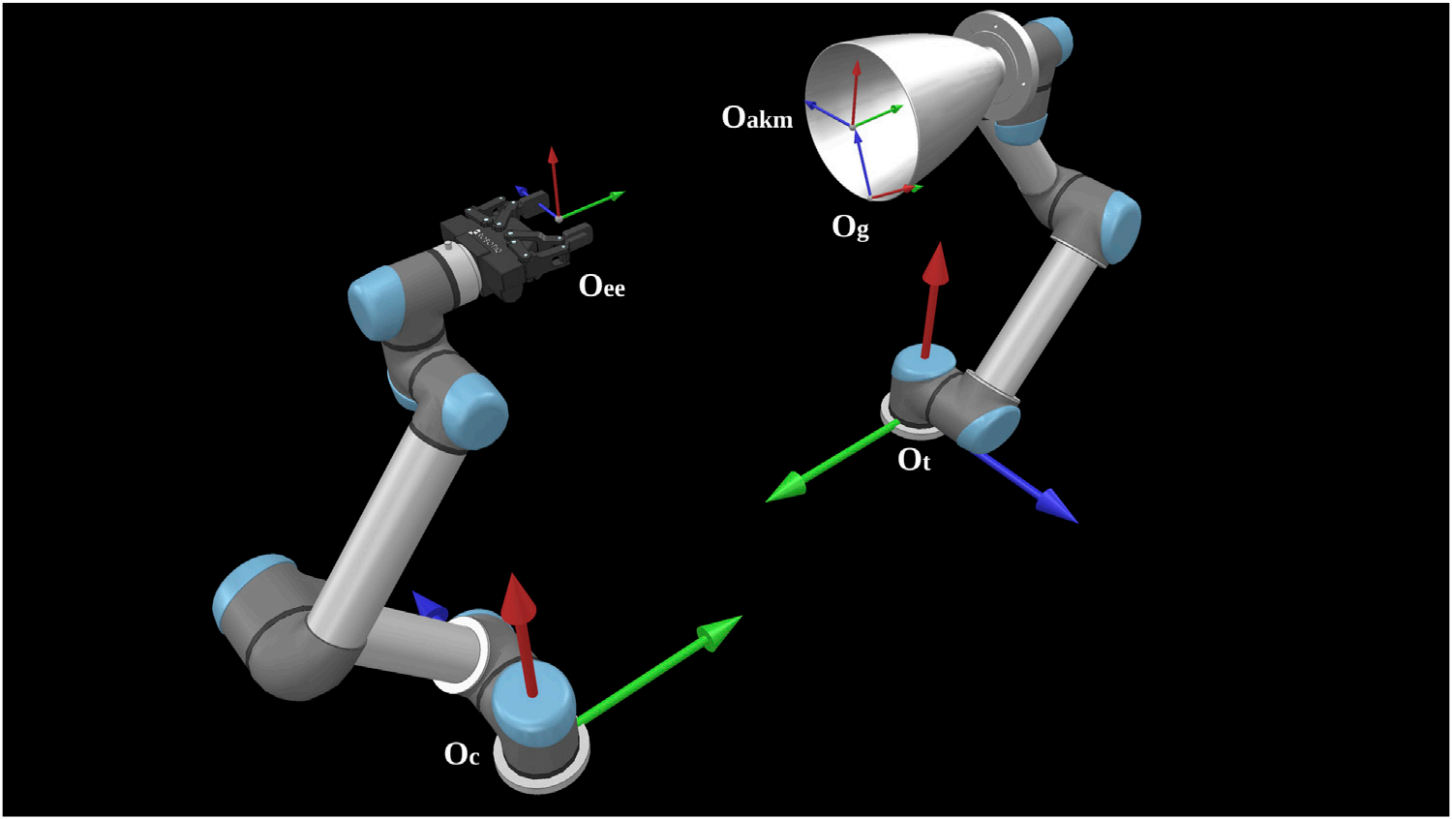
The coordinate frames used for slippage calculation for our real experiment.

In lack of a laser or tactile skin sensor, we measure the slippage not of each finger, but of the gripper end-effector coordinate frame w.r.t. the grasping point frame. This leads to each attempt having one set of measurements instead of two, as it was the case in the simulation. A schematic that shows the frames involved in the computations is shown in Figure 14. We use the following coordinate frames:

- $O_{c}$ The frame of the chasing robot’s base.
- $O_{t}$ The frame of the target robot’s base.
- $O_{ee}$ The frame attached on the end-effector (gripper’s tip) of the chasing robot.
- $O_{akm}$ The frame attached on the end-effector (nozzle tip) of the target robot.
- $O_{g}$ The grasping frame attached on the nozzle surface, right after contact with the fingers.

We note with $T_{j}^{i}$ the coordinate transformation from frame *i* to frame *j*. This means that $T_{j}^{i}$ expresses the pose of frame *j* w.r.t. the frame *i*. We have access to the following transformations:

- $T_{t}^{c}$ from past calibration of our testbed, performed with visual markers offline.
- $T_{ee}^{c}$ from the chasing UR5 control software.
- $T_{akm}^{t}$ from the target UR5 control software.

**FIGURE 15:**
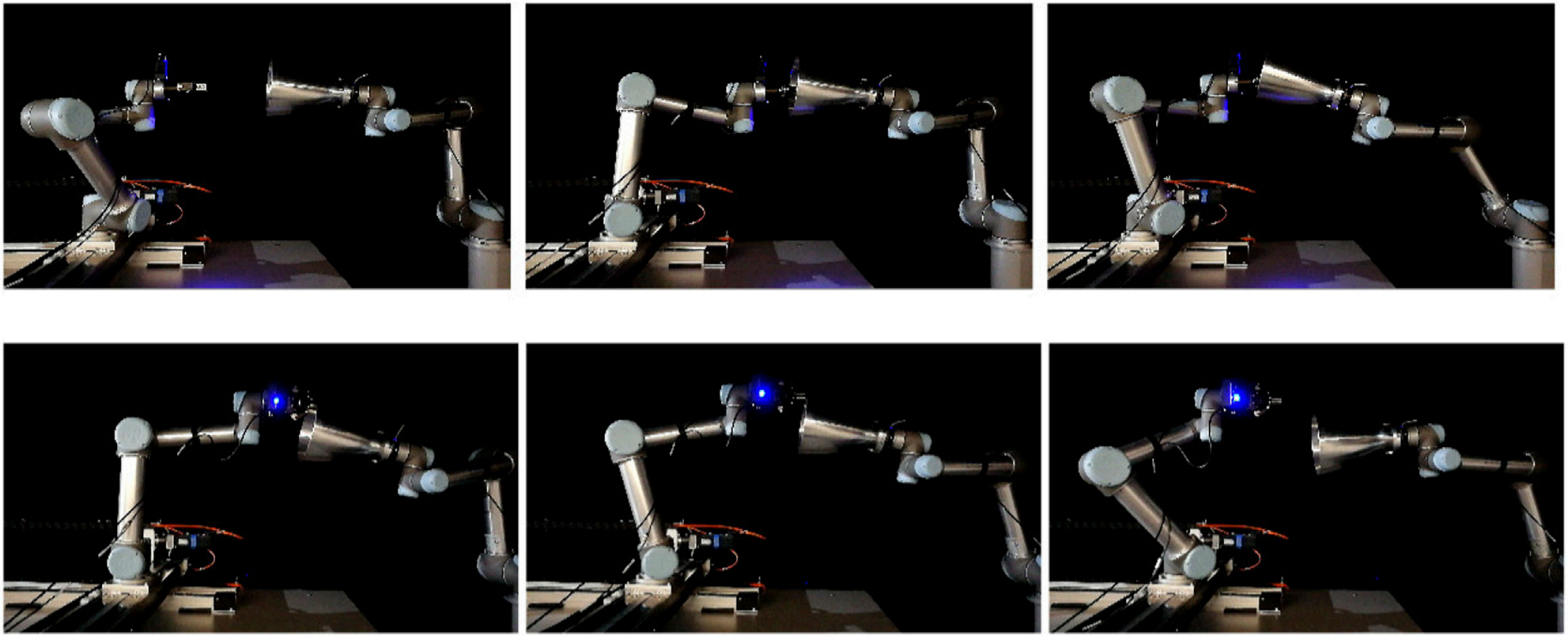
An execution sequence for a successful attempt for grasp no 6 **(top row)** and a failed attempt for grasp no 7 **(bottom row)** of the real experiment. The images show the approaching pose **(left)**, grasping pose **(middle)**, and final pose after pulling **(right)**. In the case of the successful attempt, the robot holds the target for the whole pull duration. In the failed attempt, after 3 s of pulling the gripper loses contact of the target and the grasp breaks.

**FIGURE 16:**
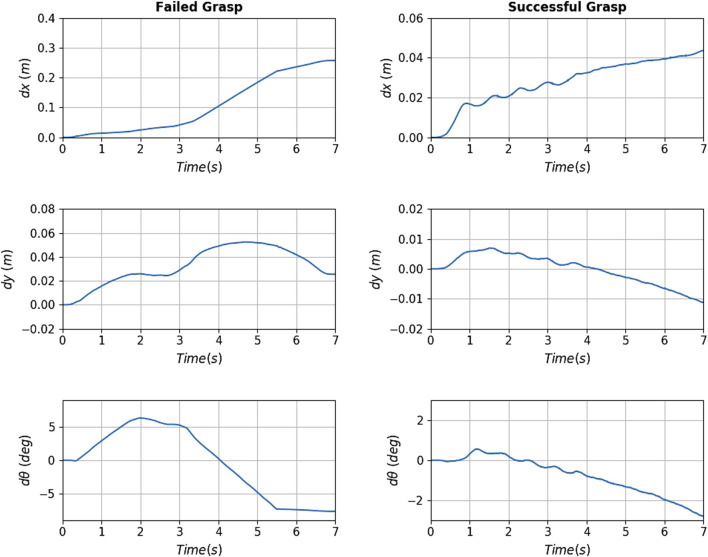
Slippage signals for the failed (**left** column) and successful (**right** column) of Figure 15. The translational slippage (**top and middle** graphs) and rotational slippage (**bottom** graphs) over the whole pull duration are shown. For the failed grasp, at about 3.3 s the robot loses contact with the target. The successful grasp manages to yield relatively low slippages, securing the capturing of the target.

The measurement process is as follows:

- We command the chasing robot to reach the target and close the gripper fingers around the grasping point. When this happens, the frames $O_{ee}$ and $O_{g}$ are identical, and we have $T_{g}^{c}=T_{ee}^{c}$.
- We read $T_{ee}^{c}$ from the forward kinematics of the chaser arm.
- We read the AKM endpoint pose $T_{akm}^{t}$ from the forward kinematics of the target arm.
- We transform $T_{akm}^{t}$ to $O_{c}$, using the calibration $T_{t}^{c}$, i.e., $T_{akm}^{c}$ = $T_{t}^{c}\text{*}T_{akm}^{t}$.
- We calculate the transformation $T_{g}^{c}$ by multiplying $T_{akm}^{c}$ and $T_{g}^{c}=T_{ee}^{c}$, i.e., $T_{g}^{c}=T_{akm}^{c}\text{*}T_{g}^{c}\Rightarrow T_{g}^{c}={\left(T_{akm}^{c}\right)}^{-1}\text{*}T_{e}e^{c}$.
- We then start the pulling motion. We measure $T_{akm}^{c}$ (that is, we measure $T_{akm}^{t}$ and apply $T_{akm}^{c}$ = $T_{t}^{c}\text{*}T_{akm}^{t}$ on every measurement), and $T_{ee}^{c}$ for the whole pull.
- This results to a set of transformations for the AKM endpoint $T_{akm,traj}^{c}=\left(T_{akm,0}^{c},T_{akm,1}^{c},\ldots ,T_{akm,i}^{c}\right),i=N$ with *N* the number of measurements that represent the AKM endpoint instances during the pulling. We transform every instance of this trajectory to the grasping point, by applying $T_{g}^{c}$. This way, we end up with the pulling trajectory of the grasping point expressed in the chaser frame $T_{g,traj}^{c}$.
- We also measure the chaser end effector trajectory $T_{ee,traj}^{c}$ at every pulling instance. The translational slippage is finally calculated as the euclidean distance between $T_{ee}^{c}$ and $T_{g}^{c}$ at every instance of the trajectory. The rotational slippage is calculated as the rotation angle in the *z* axis (perpendicular to the surface) at every trajectory instance. The result is two slippage signals for every attempt. If the gripper still retains the nozzle in the end of the pulling motion, the attempt is deemed successful, otherwise the attempt is considered failed. Figure 15 shows execution instances of the successful and failed attempt, and Figure 16 the corresponding slippage signals.

**FIGURE 17:**
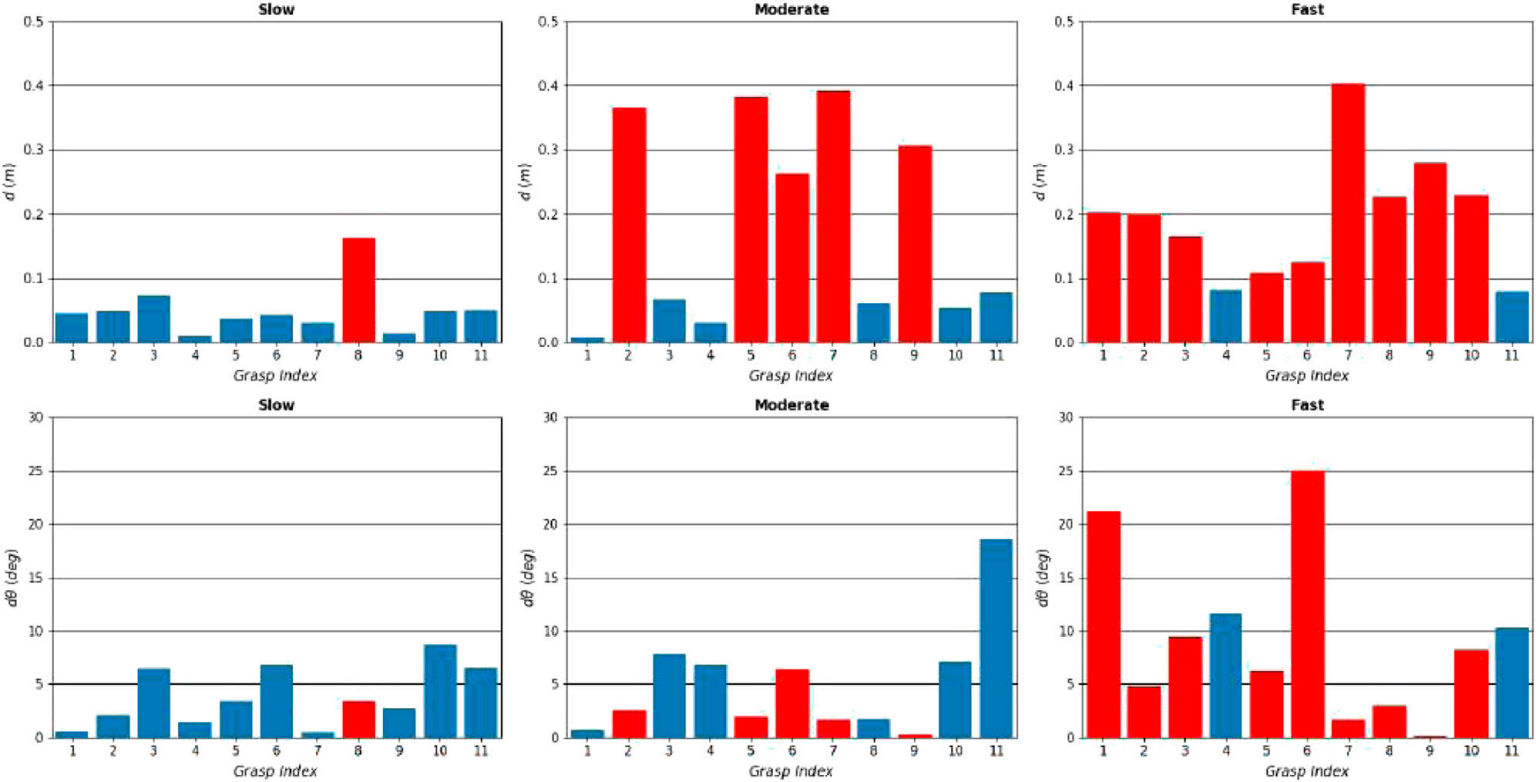
Results of the real experiment. Maximum translational and rotational slippages observed over all attempts, for slow **(left column)**, moderate **(middle column)** and fast **(right column)** velocities, plotted against the 11 grasping points. The successful and failed attempts are shown in blue and red bars respectively. The graphs show that the slower the pulling profiles is, the higher the success chance becomes.

Figure 17 shows the maximum translational and rotational slippages observed at every attempt, for slow, moderate and fast velocities. The blue bars show successful attempts, and red bar shows failed attempts. The graphs show that overall, the attempt success rate is inversely proportional to the velocity value. The slow velocity has a success rate of 90% (10 out of 11 attempts), the moderate velocity 54% (6 out of 11 attempts) and the fast velocity 18% (2 out of 11 attempts). This can be attributed not only to the increasing velocity, but also the increasing acceleration of the pulling profiles. When the acceleration is high, the increased inertia of the target play an important role in generating additional pulling loads that a grasp with given applied force has to overcome. This is not the case in slower pulls, where inertia is not so effective. In addition, in fast accelerations the pulling force can overcome the frictional loads developed on the gripper-nozzle contact easier, leading to contact sliding and increased chances of grasp breaking.

It was observed in the experiment that dx, the slippage component parallel to the bell (noted with green arrow in Figure 2) generally tends to be higher than dy, the slippage component along the circumference of the nozzle radius (noted with red arrow in Figure 2). This happens because the internal finger is more restricted by the concavity of the nozzle in the *x* direction, and the bulk of the pulling motion is directed towards the flatter −y direction, where there is limited resistance due to geometry. The AKM machined model has some minor concavity towards the −y direction due to its “expanding bell” shape as it is typical of AKMs, and even though it offers additional resistance it is generally not enough to resist fast pulls.

In most attempts, the rotational slippage is not very high (less than $10^{o}$). An interesting finding is that even in some attempts that show increased rotational slippage (over $15^{o}$) the grasp may not break. The rotational slippage merely reflects to a pivot around the grasping point. Eventually, the translational slippage, and mostly the slippage directed towards the cone exit, is the definitive factor that affects the attempt success or failure.

The results show that grasp 4 (manual) and grasp 11 (synthesised) performed the best along with all motion profiles, with no failures. This seems to suggest that grasping the target from the bottom or top of the nozzle yields lower slippage and increased chances of successful capture. This is counter intuitive, as the first seven grasps have similar local geometry, and the pulling motion direction is the same for all seven grasps. In our testbed, the top and bottom grasps were aligned with the chasing robot’s base. When the robot pulled towards the chaser base, it pulled on a straight line, while on the other grasps that were not aligned, it had to introduce a minor curve in the pulling direction. As a result, an interesting finding of the experiment is that pulls towards a colinear direction to the spacecraft base (manifested with the chasing UR5 base) did not introduce loads that tend to destabilise the grasp. This information would be used for the rendezvous and the manipulator motion planning phases, to ensure alignment between the spacecraft’s centre of mass and the detected grasping point on the target.

Finally, the grasping force was set to 150 N, however in reality it varies according to the contact point position on the gripper pad, and whether the two pads meet the target in an antipodal and parallel way. This is a limitation of our RobotiQ gripper, due to its finger pivoting structure and minor rotational compliance of finger pads. We could not control these parameters in our experiment, and some grasps failed mostly due to initial incorrect positioning of the gripper that resulted in significantly lower applied force. For real applications, continuous application of the desired applied force needs to be ensured with innovative grasping mechanisms.

### 4.3 Sensitivity Experiment

The previous experiments tested the efficacy of grasping an engine nozzle with a robotic gripper. In order to test the variation in the grasping behaviour when the parameters that affect stability are changed, we wet up a sensitivity experiment using our real robot testbed. This experiment serves as experimental validation of the numerical analysis we performed in Mavrakis and Gao (2020). The goal of the experiment is to determine whether the theoretical results presented in Mavrakis and Gao (2020) are valid when tested under realistic conditions.

**FIGURE 18:**
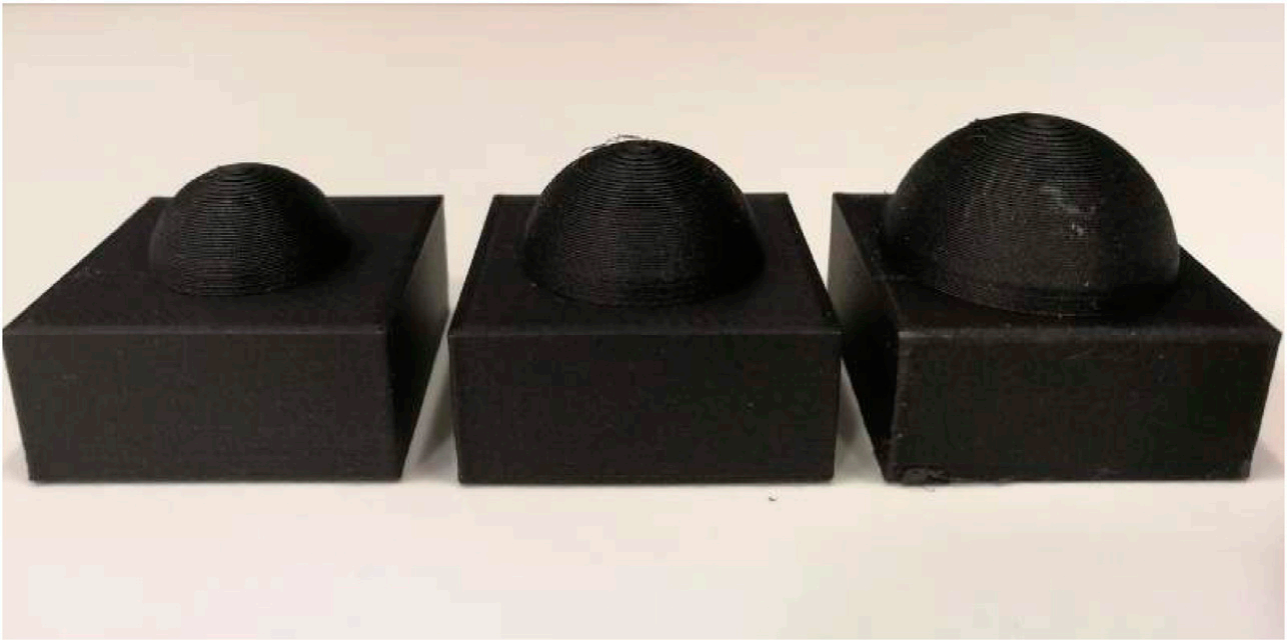
The three spherical fingertips used for the sensitivity experiment. Each tip was printed twice and mounted on the gripper. From left to right: $r_{f}=0.008,0.01,0.012m$.

In this experiment, we define a capturing scenario of a STAR-24c target. We manually place the gripper on a grasping point at the bottom part of the metallic nozzle, and we use this grasping configuration as our main contact point. As the analysis in Mavrakis and Gao (2020) was performed for spherical fingertips, we manufactured three sets of 3D-printed spherical fingertips, which we mounted on the Robotiq gripper. The fingertips are shown in Figure 18. The fingertips have radii $r_{f}$ of 0.008,0.01 and 0.012m. The radii were selected to match the $r_{f}$ of the theoretical analysis ($r_{f}=0.01m$) and variations of ±0.002m.

The parameters we examine are the grasping force $F_{n}$, finger radius $r_{f}$, nozzle radius *r*, and contact yaw angle *ψ*. As with our theoretical analysis, we define a set of *nominal* capturing parameters. These nominal parameters are given in Table 1.

**TABLE 1:** Nominal parameter values.

Parameter	Value
$F_{n}\left(N\right)$	180
$\psi {(}^{o})$	0
$r_{f}\left(m\right)$	0.01
r(m)	0.04

We command the robot to reach and grasp the target from the grasping point, and execute the same pulling motion as in our previous experiment. We change one grasping parameter each time, keeping all others at their nominal values. The variation of each parameters is conducted according to our theoretical analysis, examining parameters both greater and lower than the nominal. As we were not able to measure exact nozzle radii, the nozzle radius *r* given in Tables 1, 2 refer to distance from the nozzle edge towards the nozzle interior. A point that is towards the interior yields a smaller nozzle radius, even though it is noted with a larger distance variable on the Tables.

**TABLE 2:** Success rates for the sensitivity experiment.

Parameter	Value	Success %	Difference from nominal
$F_{n}\left(N\right)$	140	40	−20
–	160	20	−40
–	200	60	0
–	220	80	20
$\psi {(}^{o})$	−20	60	0
–	−10	40	−20
–	10	40	−20
–	20	40	−20
$r_{f}\left(m\right)$	0.008	20	−40
	0.012	80	20
–	0.01	0	−60
–	0.07	80	20
Nominal	–	60	–

**FIGURE 19:**
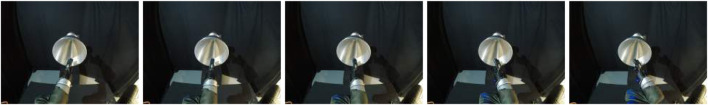
The five *ψ* angles used for the sensitivity experiment. From **left to right:** $\psi =-20^{o},-10^{o},0^{o},10^{o},20^{o}$.

For the angle *ψ*, we set up the range based on a safe limit that the robotic gripper can rotate without hitting the nozzle edge with the wrist. This range was found to be $\left[-20^{o},20^{o}\right]$, and we selected $10^{o}$ interval, so we have five grasps, including the nominal one that yields $\psi =0^{o}$. The five grasping points are shown in Figure 19.

For each parameter variation we execute five grasping attempts, and note the grasping result (success or failure). We then use the success percentage over these five grasps as evidence for the grasp stability. The results are given in Table 2.

The results suggest a confirmation of the theoretical results presented in Mavrakis and Gao (2020). As with the theoretical results, the grasps are stabler and the success rate increases when the grasping force from the fingers increases. This is also an intuitive result, as stronger grasping forces tend to increase the frictional forces required to break the grasp. The variation in *ψ* angle does not seem to have a definitive effect on the grasp stability, with the success percentages remaining similar. This happens because of the uniform and equal curvature of the spherical fingertips, that remains unaffected under rotations of the contact frame. The most interesting results are provided by the fingertip and nozzle radii variations. As the fingertip radius increases, the contact area with the nozzle increases, and hence the frictional loads that the grasp can withstand. As a result, the grasps become substantially stabler with higher success rate. In contrast, a lower fingertip radius yields a smaller contact area that becomes more point-like. This leads to lower success rate. Similarly, A larger nozzle radius (with a constant fingertip radius) yields a zero success rate, and a smaller nozzle radius results in increased stability. The outcome of this experiment seem to re-affirm the necessity of using high, constantly applied forces for capturing and a ratio $\frac{r_{f}}{r}\leq 1$, as close to 1 as possible.

We do not use the slippage measurements for this experiment, as they did not provide any additional information that the other two experiments did not show. Again, the results with higher slippage tended to break the grasp and decrease the success rate, which is an outcome studied and confirmed by the previous two experiments.

It should be noted that an additional property that should be included in the sensitivity analysis was the friction coefficient of the contact. This would require the manufacturing of fingertips with same radius and different materials, something that was not possible at the time of our experiments. In addition, the repetitive dragging of the fingertips to the nozzle tended to slowly degenerate the surface, further changing the friction coefficient. Under control of such conditions, a more elaborate experiment that shows the effect of the friction coefficient on the grasp stability would be an interesting extension for this study.

## 5 Discussion

The presented work serves as a preliminary analysis and testing of how existing robotic grasping research could be transferred to space robotics. While the overall results are promising, it is useful to discuss them more analytically to highlight merits and shortcomings.

### 5.1 Dependence on Physical Parameters

The developed stability criterion depends on the knowledge of frictional and elastic properties of the materials in the contact. These parameters are difficult to calculate, and they are also prone to change due to environmental conditions, which can change dramatically in orbit. While their variation has an overall effect on the eigenvalues of the ISM, our previous analysis [Mavrakis and Gao (2020)] has shown that they tend to affect the larger eigenvalues. As a result, the *minimum eigenvalue* criterion remains virtually unaffected by frictional and elasticity variations. Even so, as increased friction and elasticity tend to stabilise the grasp, it is preferable to select the fingertip materials with the intention of producing a softer, high-friction contact with the target.

Similarly, the inertial parameters of the target were used for the minimum eigenvalue criterion. They are generally known in advance for operating targets, but not necessarily for pieces of space debris. Determining them is more straightforward than in terrestrial robotics, as the targets are typically free-floating and rotating, contrary to objects on earth that are in rest. This can be done before grasping [Christidi-Loumpasefski et al. (2017)] to have an initial estimate about the stability, or post-grasping [Ekal and Ventura (2020)] to regulate the estimation and compensate by applying additional force if needed.

### 5.2 Mechanism Stiffness

The ISM criterion assumes that the finger mechanism is perfectly stiff. We wished to have hardware-independent analysis, and so we assumed perfect stiffness to distinguish between the effect on stability resulting from the *grasp geometry*, and the effect resulting from the *finger mechanism*. In reality, all robotic fingers have an infinitesimal compliant behaviour. As mentioned, the equivalent of the ISM for compliant manipulators is the Grasp Stiffness Matrix and it should be used in real robots, especially with bespoke grippers and hands. Mechanisms and control algorithms for variable stiffness are also useful. This way the robot can achieve low stiffness in the contact phase, ensuring little disturbance to the target, and high stiffness in the rigidisation and pulling phase, ensuring a strong grip. In the real testbed experiment, the stiffness of the robotic gripper was affected by the positioning of the fingers and the configuration of the freely-titling finger pad on the surface. In some cases, especially in faster motion profiles, the stiffness was not able to properly apply 150 N, leading to increased slippage and breaking of contact. Our presented work requires hardware and control algorithms able to maintain stiffness constant force application, that has been shown to greatly affect stability [Mavrakis and Gao (2020)].

### 5.3 External Disturbances and Stability Limits

The results from the real testbed experiment suggest that pulling the target with low velocity and acceleration yields better chances to secure the object than pulling with a high velocity-acceleration profile. This agrees with intuition, as pulling an object slower reduces the effect of the inertial forces resisting the grip. The theory of ISM was developed to correspond to smaller, lighter objects that can be enclosed by the gripper counteracting their weight. The inertial forces were discussed as minor disturbances in the finger positioning, well withing the stability limits of the grasp. However, orbital targets are typically large and heavy, and so tend to produce higher inertial forces that can dislocate the finger pads and break the grasp. In addition, the rigidisation phase of orbital capturing leads to various disturbances resulting from thrusting to stop tumbling, as well as the gradual increase of manipulator stiffness. The limits of the stability w.r.t. the orbital operations and the disturbances they inflict on the grasp need to be analysed and tested further.

### 5.4 Control Strategies

We conducted all of our motions (chasing arm and gripper) using position control for the positioning of the gripper on the target grasp and the closing of the gripper. Position control offers placement accuracy, at the expense of controlling the applied force to avoid contact disturbances. This was beneficial for our experiments as we wished to see whether the natural curvature of the nozzle bell would assist the capturing motion. Indeed, both in simulation and real experiment, the interior finger slowly “slid downhill” towards the concave part of the nozzle bell in all grasps, with the exterior finger following and resting on the antipodal part of the bell. While it was positive to confirm that grasping from concave parts increase stability, in reality space robots need to perform the same capturing motions with some form of compliance control. Such controllers trade positioning accuracy for the ability to regulate the contact force, however by aiming for concave surfaces of the target they can also induce this “sliding” to the concave part and increase the grasp stability. This outcome from our paper serves as guidance for future grasp planners for orbital robots.

## 6 Conclusion

In this paper, we presented a methodology for capturing a piece of space debris, namely the nozzle of a spent upper rocket stage, with a robotic antipodal gripper. We drew inspiration from existing research on robotic grasping, to evaluate the stability of a robotic grasp on the nozzle bell. We claim that since stability is a property that dictates the robustness of a grasp under external forces, it could be applied in place of the mechanically-locking mechanisms used for orbital target capturing. To evaluate this claim, we set up a simulation and two experiments with a real testbed. In simulation, we set up a scenario of a spacecraft with a robotic arm a gripper capturing and pulling three different apogee kick motor models by their nozzle, under four grasping points and three motion velocities, for three physics engines. The measured slippages suggest that the grasps are quite stable among all targets, engines, grasps, and pulling velocities. In the first real testbed experiment, we set up a robotic arm capturing and pulling a target, under 11 grasps and three pulling profiles. The results suggest that the stability decreases when the pulling velocity and acceleration are increased. In the second real testbed experiment, we perform a sensitivity analysis to evaluate the stability variation under variation of each grasping parameter at a time. The results seem to agree with the numerical analysis of our past work.

The presented work can be extended in numerous ways. The grasp stability analysis can be extended to multi-fingered hands, which have the advantage of dexterous positioning of the fingers in the surface to minimise slippage. This would lead to the development of methodologies for multi-fingered grasp synthesis and planning for space robotics, a field with little existing research.

It should be noted that while in robotic grasp stability and synthesis research, it is typical to test on objects of various geometries to determine the algorithm capabilities, in this paper we base our analysis over the stability of the *finger-nozzle bell* grasp only. Introducing new target geometries would be out-of-scope for this paper. However, examining the applicability of the ISM on other orbital targets is an interesting future research opportunity.

Our presented testbed has significant advantages, but also limitations such as the physical workspace and range of operations. The experimental results could be also correlated with more advanced weightlessness testbeds, with the intention of eventually testing them on-board a real mission.

Finally, to increase the applicability of our methodology, the presented analysis could be carried out for other targets and spacecraft parts such as Marman rings for spacecraft docking, rocks for surface sampling, and tools for operations onboard the ISS.

## Data Availability

The raw data supporting the conclusion of this article will be made available by the authors, without undue reservation.
